# Conservation of imprinting of *Neuronatin* (*Nnat*) in rabbits

**DOI:** 10.1186/s40064-015-1054-z

**Published:** 2015-06-13

**Authors:** Feifei Duan, Xianju Chen, Lin Yuan, Yuning Song, Anfeng Wang, Qingyan Lv, Zhanjun Li, Liangxue Lai

**Affiliations:** College of Animal Science, Jilin University, 5333#, Xi’an Road, Changchun, 130062 China

**Keywords:** DMR, Imprinted gene, *Nnat*, Paternally expressed, Promoter, Rabbit

## Abstract

Although the expression and epigenetic status of imprinted genes have been extensively studied in a number of species, less is known about the genomic imprinting in rabbits. *Neuronatin* (*Nnat*) plays significant roles in the brain development and metabolic regulation and has been identified to be imprinted and paternally expressed in humans, mice and pigs; however, it has not yet been investigated in rabbits. In this study, we confirmed the expression of two isoforms of the rabbit *Nnat* (*Nnat*-a and *Nnat*-β) identified in Genbank and Ensembl by quantitative real-time PCR. In addition, we also determined the methylation profile of the CpG island in the promoter region of the rabbit *Nnat* using bisulfite sequencing PCR and combined bisulfite restriction analysis. Here, we provide the first evidence that *Nnat* has two transcripts in rabbit. Additionally, the CpG island located in the promoter region shows oocyte-specific methylation and may be the differentially methylated region of *Nnat* in rabbits.

**Electronic supplementary material:**

The online version of this article (doi:10.1186/s40064-015-1054-z) contains supplementary material, which is available to authorized users.

## Background

The model organisms play an important role in the study of human diseases. Owing to the unique features of the lipoprotein metabolism and being sensitive to cholesterol diet, rabbit models have been widely used to study human atherosclerosis, and have also made a great contribution to the translational research (Fan et al. 2014). The genomic imprinting phenomenon is theorized to exist in all the placental mammals and plays an important role in the regulation of fetal growth, development, and postnatal behavior, however, little is known about imprinted genes in mammalian species apart from human and mouse (Zhang et al. 2012; Dindot et al. 2004). At present, 80 imprinted genes have been identified in humans, 125 in mice, but only 20 in cows, 16 in sheep and just one imprinted gene (*Impact*) was confirmed in rabbits (http://igc.otago.ac.nz/home.html) (Zhang et al. 2012).

The *Nnat* was discovered in the neonatal rat brain for the first time and was subsequently found to play a significant role in the neuronal development, it is maternally imprinted in humans, mice, cattle and pigs (Evans et al. 2001; Kagitani et al. 1997; Zaitoun and Khatib 2006; Cheng et al. 2007). The more recent data have also demonstrated that *Nnat* participates not only in neuronal growth, but also in pituitary development, glucose-mediated insulin secretion in the pancreas and keratinocyte differentiation in the skin (Joseph 2014; Chen et al. 2014). Additionally, the *Nnat* protein has been also found to be located in the aortic endothelium which may increase endothelial cell adhesion molecule expression mediated through phosphatidylinositol 3-kinase (PI 3-kinase)/p38-dependent activation of nuclear factor-κB (NF-κB). Therefore, *Nnat* is thought to be a candidate molecule that might be involved in inflammatory pathways associated with obesity and insulin resistance related to the endothelial dysfunction and/or the development of atherosclerosis (Mzhavia et al. 2008). All of the above findings suggest that *Nnat* plays a number of important roles in the mammalian development.

Although *Nnat* is known to be imprinted in many species, its imprinting status has not been determined in rabbits. In this study, we aimed to determine the expression levels of the rabbit *Nnat* in the brain, liver, kidney, eye and fetus by quantitative real-time PCR (qPCR). The bisulfite sequencing PCR (BSP) and combined bisulfite restriction analysis (COBRA) were performed to further determine the imprinting status and differentially methylated region (DMR) of *Nnat* in different rabbit tissues including the brain, liver and germ cells.

## Results and discussion

**Figure 1:**
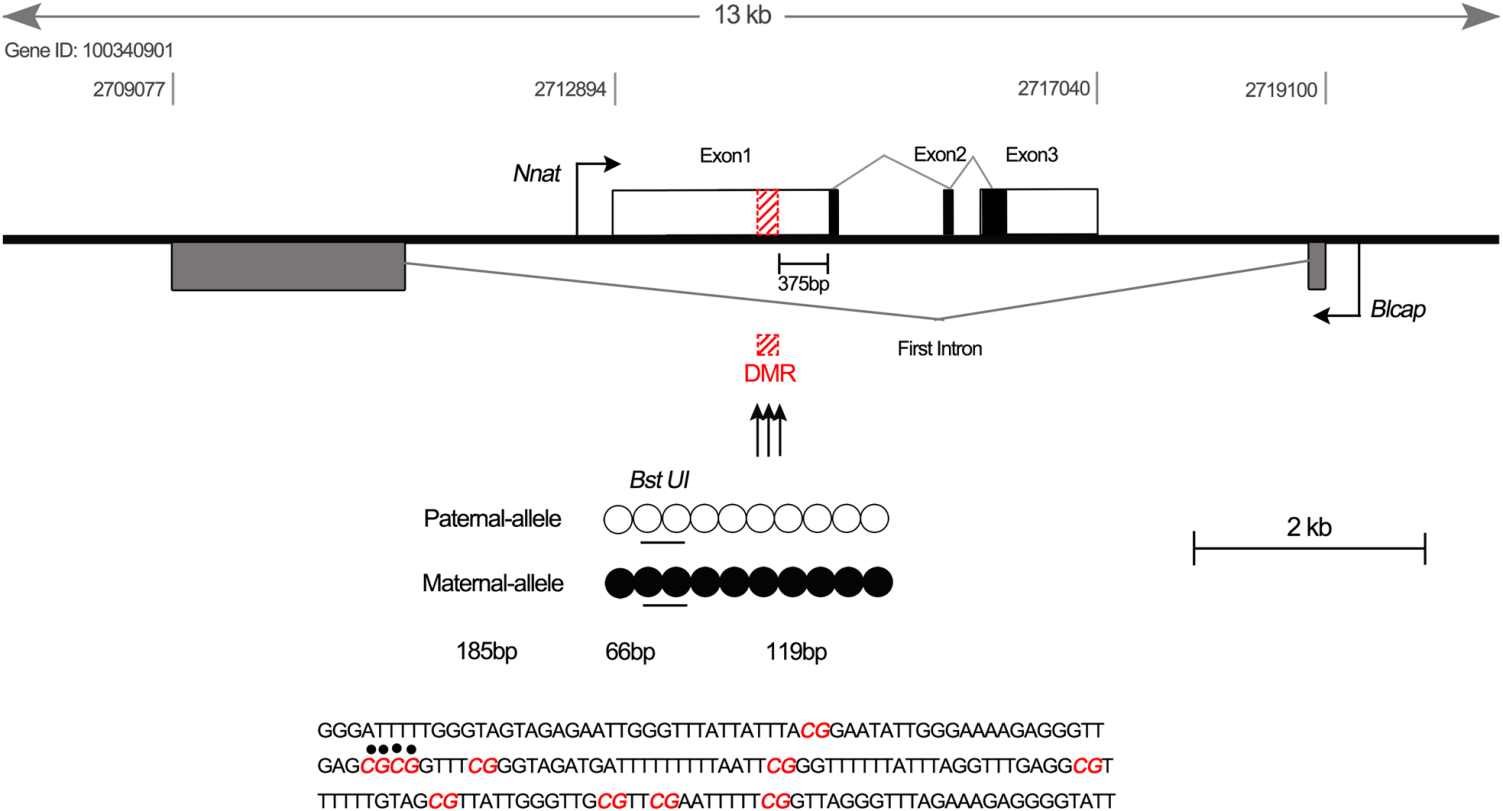
Structure of the *Nnat* locus in rabbits. The *Nnat* is located within the intron of *Blcap* and consists of three exons (*white* and *black rectangular boxes*) and two introns. The protein coding regions are indicated as *black boxes*. The *gray boxes* indicate *Blcap*. The *black arrows* indicate the transcription start sites of *Nnat* and *Blcap*. The *box* with *red stripes* within the promoter of *Nnat* represents the CpG island within the amplified region of BSP, which might be the DMR of the *Nnat* in rabbits. The *circles* indicate individual CpG dinucleotides within the BSP. Unmethylation (*open*) in the paternal-allele and methylation (*closed*) in the maternal-allele were presumed. Bisulfite-converted sequence of BSP products is shown with each CpG site marked in *red*; BstUI restriction sites (CGCG) are shown as *black solid dots* above the CpG sites and as *bars* below the *circles*.

**Figure 2:**
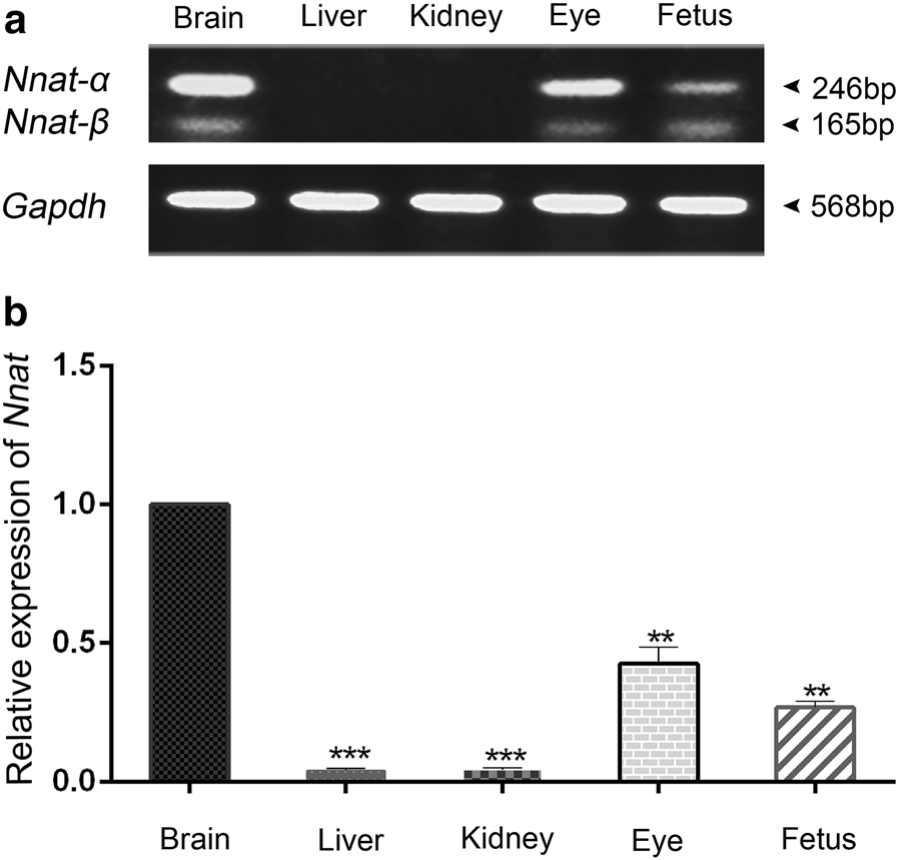
The expression levels of rabbit *Nnat* in different rabbit tissues. **a** RT-PCR detection of the *Nnat* α-form (246 bp) and β-form (165 bp) in brain, liver, kidney, eye and fetus. *Gapdh* was used as control for RNA/cDNA integrity. **b** The comparison of *Nnat* expression levels in brain, liver, kidney, eye and fetus by qPCR. *Gapdh* was used as a reference gene. Data are shown as mean ± SEM (n = 6). **(p < 0.01) and ***(p < 0.001) indicate statistically significant differences.

The *Nnat*, which is a highly conserved gene among different species including humans, cattle and pigs, is located within the 8.5-kb intron of the *Blcap* (Bladder Cancer-Associated Protein) gene and contains three exons and has two alternatively spliced transcripts (α and β) (Schulz et al. 2009; Cheng et al. 2007). To further understand the expression pattern of *Nnat* in rabbit, primers were designed according to the two alternatively spliced transcripts (*Nnat*-α and *Nnat*-β), which encode 81 and 54 amino acid proteins, respectively (Figure 1). RT-PCR and qPCR results demonstrated that both *Nnat*-α and *Nnat*-β are expressed in brain, eye and fetus of rabbit. Consistent with the EST-derived gene expression data, both the transcripts were highly expressed in brain but not expressed in liver and kidney (Figure 2). In contrast to rabbit, two transcripts of *Nnat* are widely expressed in most of tissues including liver and kidney in cattle (both fetal and adult) and in 2-month pig (Cheng et al. 2007; Zaitoun and Khatib 2006), which means that there are different *Nnat* expression profiles among different species. As *Nnat* is thought to play a number of important roles in the mammalian development, it would be very useful to identify the expression patterns and the imprinting status of the *Nnat* in rabbits in order to analyze the conservation of genomic imprinting among different species.

**Figure 3:**
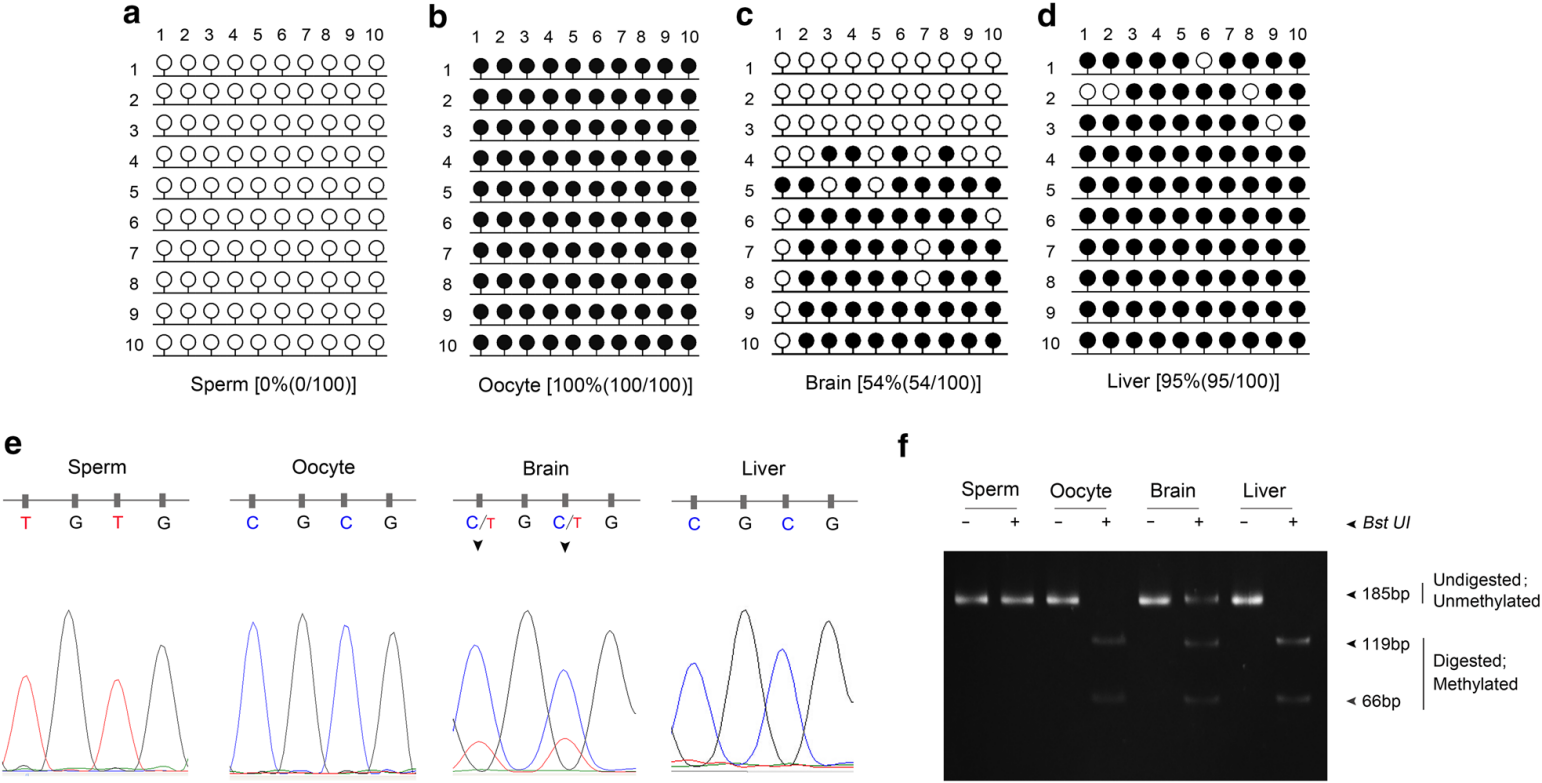
The methylation status of the *Nnat* promoter region in rabbits. The methylation status was analyzed in sperm (**a**), MII oocytes (**b**), brain (**c**) and liver (**d**) by BSP. The *open* and *closed circles* indicate unmethylated and methylated CpG sites, respectively. The *number* in the *parentheses* represents the methylated CpG sites relative to the total CpG sites counted. **e** The sequencing analysis of the CpG island within the *Nnat* promoter in BstUI recognition sites in the sperm, MII oocytes, brain and liver. *Inverted black triangles* indicate differentially methylated cytosine. **f** COBRA analysis of the products of BSP, which were digested with (+) or without (−) restriction enzyme BstUI in sperm and MII oocytes, brain and liver. Fully methylated CpG sites are represented by two DNA fragments (66 and 119 bp) and unmethylated CpG sites are represented by one DNA fragment (185 bp).

Previously we have shown that the *Nnat* promoter sequence is a highly conserved region and the expression of the *Nnat* is significantly associated with the methylation status of the CpG island located in the *Nnat* promoter sequence in the pigs (Chen et al. 2014). To identify whether the promoter was involved in the regulation of *Nnat* expression in rabbits, the DNA methylation profile of the promoter CpG island located 375 bp upstream of the first ATG codon in exon 1 was determined in the sperm and MII oocytes (Figure 1). The data of BSP showed unmethylated and fully methylated promoter region of the *Nnat* in sperm (Figure 3a) and MII oocytes (Figure 3b), respectively. Similar findings were observed using the COBRA analysis in both samples (Figure 3f). Some epigenetic information must be differentially contributed from the two gametes, including known imprint control regions (ICRs) that maintain their allele-specific methylation pattern throughout embryogenesis (Smith et al. 2012). The gamete-specific methylation pattern of rabbit *Nnat* was consistent with our previous study which suggested that imprinting marks of *Nnat* were established via a sex-specific mechanism (Chen et al. 2014). Generally, the DNA methylation marks are established at zygote and primordial germ cells with a unique set of mechanisms regulating the DNA methylation erasure and re-establishment (Seisenberger et al. 2013). These findings suggested that the *Nnat* might be a paternally expressed gene in rabbits.

To further confirm the imprinting status of the rabbit *Nnat*, methylation profiles of the brain and liver tissues with or without the expression of *Nnat* were analysed using BSP and COBRA. The results showed that both methylated and unmethylated strands were obtained, in the identified CpG island in brain but hypermethylation was determined in liver (Figure 3c–f), indicating that the maternal allele might be methylated and the paternal allele unmethylated in a tissue-specific manner (Figure 1). The above findings suggest that *Nnat* is likely to be a paternally expressed gene, which is consistent with the previous reports on humans, mice, cattle and pigs (Evans et al. 2001; Kagitani et al. 1997; Zaitoun and Khatib 2006; Cheng et al. 2007). Additionally, the partial methylation and hypermethylation of the *Nnat* promoter observed in rabbit brain and liver tissues, respectively, demonstrated that the *Nnat* gains methylation in non-expressing tissues. And the gamete-specific methylation pattern revealed the characteristics of an imprinted gene and identified the CpG promoter region being a putative DMR of the *Nnat* in rabbits.

## Conclusions

Here, two transcripts of the *Nnat* in rabbit have been determined and the putative DMR located in the promoter sequence of *Nnat* in rabbits is identified for the first time. The gamete-specific methylation pattern of rabbit *Nnat* suggests that the *Nnat* is likely to be a paternally expressed gene in rabbits. In conclusion, we deduced that the conserved imprinting patterns among humans, mice, cattle and pigs suggest similar functions of *Nnat* in different mammalian species.

## Methods

### Ethics statement

The experimental procedures involving model organisms (rabbits) were performed in accordance with the specified guidelines on the animal care and the use of animals in research, which were approved by the Animal Care and Use Committee of the Jilin University, Changchun, China.

### Sample collection

The brain, liver, kidney and eye were collected from 6-month old New Zealand White rabbits and normally fertilized fetuses were collected on Day 22 of gestation. All the samples were immediately stored in the liquid nitrogen till further use. MII oocytes were collected as described in a previous report (Tian et al. 2012). Briefly, sexually mature rabbits were superovulated by injecting six successive intramuscular doses of 60 IU of follicle-stimulating hormone (FSH; from Ningbo Second Hormone Factory) every 12 h and then intravenous injection with 100 IU of human chorionic gonadotropin (hCG; from Ningbo Second Hormone Factory) after 12 h to induce ovulation. The cumulus-oocyte complexes (COCs) were flushed and collected from the oviducts at 13–14 h after the hCG injection. Mature oocytes were isolated from the COCs by 100 IU⁄ml hyaluronidase in M199. The pools of 100 MII oocytes were frozen at −80°C for further use in our study. Sperm were isolated using swim-up procedure. The purity and quality of the isolated germ cells were determined as described by an earlier report (Chen et al. 2014).

### Reverse transcription PCR (RT-PCR) and qPCR

RT-PCR and qPCR were carried out as previously described (Chen et al. 2014). The primer sequences and PCR amplification conditions are listed in Additional file 1: Table S1. The relative gene expression normalized to the *Gapdh* was determined by 2^−ΔΔCT^ formula. All gene expression experiments were performed three times. The detection of gene expression was presented as mean ± SEM (n = 6). The data were analyzed using student’s *t* tests with SPSS 16.0 software (SPSS Inc., Chicago, IL, USA) and *p* < 0.05 was considered statistically significant.

### BSP and COBRA

Bisulfite conversion and COBRA were performed according to previous study (Chen et al. 2014). Briefly, for bisulfite modification, genomic DNA from different tissues and germ cells was treated with the CpGenome™ Turbo Bisulfite Modification Kit (Millipore) and EZ DNA Methylation-Direct TM Kit (Zymo Research), respectively. Primer sequences used for BSP are described in Additional file 1: Table S1. The PCR products were subjected to T vector cloning (positive clones, n = 10) and sequencing analysis, which showed heterozygosity at the (C/T) peak of *Nnat*. BSP products were also digested by a restriction enzyme BstUI (Thermo Scientific, MA, USA) for COBRA analysis. DNAMAN (LynnonBiosoft) and the online software tools MethOrimer and BiQ Analyzer were used for methylation analysis in this study.

## Additional files

Additional file 1:
**Table S1.** Primer sequences for RT-PCR, qRT-PCR and BSP.

## Abbreviations

*Nnat*: *Neuronatin*
PCR: polymerase chain reaction
qPCR: quantitative real-time PCR
BSP: bisulfite sequencing PCR
COBRA: combined bisulfite restriction analysis
PI 3-kinase: phosphatidylinositol 3-kinase
NF-κB: nuclear factor-κB
DMR: differentially methylated region
FSH: follicle-stimulating hormone
hCG: human chorionic gonadotropin
COCs: cumulus-oocyte complexes
RT-PCR: reverse transcription PCR
*Blcap*: Bladder Cancer-Associated Protein
ICRs: imprint control regions.
