# Supplementary material for: Conservation of imprinting of Neuronatin (Nnat) in rabbits
Source: Springerplus. 2015 Jun 13;4:257. doi: 10.1186/s40064-015-1054-z (PMC4467822; doi:10.1186/s40064-015-1054-z)
Supplement: Additional file 1: — Table S1. Primer sequences for RT-PCR, qRT-PCR and BSP. [file 40064_2015_1054_MOESM1_ESM.docx]

**Table S1:** primer sequences for RT-PCR, qRT-PCR and BSP

| Primer |  | Sequenc­e (5’ to 3’) | T_ann_ (℃) | Product size (bp) |
| --- | --- | --- | --- | --- |
| *Nnat* | RT-F | TACATCTTCCGCGTGCTGCTG | 54 | α-form 246 |
|  | RT-R | GGGAAAGGGATCTGGTCATCAT |  | β-form 165 |
| *Gapdh* | RT-F | CGTGAACCACGAGAAGTATGA | 54 | 568 |
|  | RT-R | CCTGTTGCTGTAGCCAAATTC |  |  |
| *Nnat* | Q-F | ACTTGCCAAGGTCAGTGAG | 58 | 75 |
|  | Q-R | GGAAAGGGATCTGGTCATCAT |  |  |
| *Gapdh* | Q-F | ATCCATTCATTGACCTCCACTAC | 58 | 58 |
|  | Q-R | GTACTGGGCACCAGCATCAC |  |  |
| *Nnat* | Out-F | GGTAGAGGTTGAAAGGATTTGG | 54 | 476 |
|  | Out-R | CCCCTTCCAAAAAATTCCGCCT |  |  |
| *Nnat* | In-F | GGGATTTTTGGGTAGTAGAGAATT | 54 | 185 |
|  | In-R | AATACCCCTCTTTCTAAACCCTAAC |  |  |

T_ann_ is the abbreviation of annealing temperature. RT (The primers for RT-PCR), Q (The primers for qRT-PCR), Out (outside primers of the BSP), In (inside primers of the BSP).
